# Systemic IgA Vasculitis With Leukocytoclastic Features in a Dialysis-Dependent Patient With Primary IgA Nephropathy

**DOI:** 10.7759/cureus.106010

**Published:** 2026-03-27

**Authors:** Sona Sharma, Michelle Raja

**Affiliations:** 1 Internal Medicine, Icahn School of Medicine at Mount Sinai, Queens Hospital Center, New York City, USA; 2 Medicine, College of Osteopathic Medicine, New York Institute of Technology, Old Westbury, USA

**Keywords:** adult iga vasculitis, dialysis intolerance, end-stage renal disease (esrd), gastrointestinal bleeding (gib), iga nephropathy (igan), palpable purpura, small-vessel vasculitis

## Abstract

IgA vasculitis (IgAV) and IgA nephropathy (IgAN) are increasingly recognized as part of a disease spectrum sharing common pathogenic mechanisms involving galactose-deficient IgA1. While IgAN represents kidney-limited disease, IgAV is the systemic manifestation. The development of systemic IgA vasculitis in patients with established end-stage renal disease (ESRD) secondary to IgA nephropathy is rarely reported and presents unique diagnostic challenges. We present a case of a 51-year-old woman with ESRD secondary to IgA nephropathy on maintenance hemodialysis who presented with acute anemia, abdominal pain, arthralgias, and gastrointestinal bleeding. On hospital day three, she developed palpable purpura on the bilateral lower extremities. Skin biopsy demonstrated leukocytoclastic vasculitis with perivascular IgA, IgM, and C3 deposition on direct immunofluorescence, confirming IgA vasculitis. Treatment with high-dose systemic corticosteroids resulted in the resolution of cutaneous and systemic manifestations. This case highlights the diagnostic complexity of adult IgA vasculitis in ESRD patients with underlying IgA nephropathy, where symptoms may mimic common dialysis-related complications. Recognition of the shared pathophysiology between IgAN and IgAV is essential for appropriate diagnosis and management.

## Introduction

IgA vasculitis (IgAV), formerly known as Henoch-Schönlein purpura, is a small-vessel vasculitis characterized by IgA immune complex deposition affecting the skin, joints, gastrointestinal tract, and kidneys [1,2]. IgA nephropathy (IgAN) represents the kidney-limited form of IgA-mediated disease. While these conditions were historically considered separate entities, emerging evidence demonstrates that they share a common pathophysiology involving galactose-deficient IgA1 (Gd-IgA1), suggesting that they represent different manifestations along a disease spectrum [3,4].

IgAV is considered the systemic form of IgAN, with both diseases sharing similar geographic distributions, genetic variants, and pathogenic mechanisms, as explained by the four-hit hypothesis [4]. On kidney biopsy, IgAV-associated nephritis (IgAVN) and IgAN are histopathologically indistinguishable [1]. The key distinguishing feature is the presence of extrarenal manifestations in IgAV, particularly the characteristic palpable purpura, arthralgias, and gastrointestinal involvement [5].

The development of systemic IgA vasculitis in patients with established end-stage renal disease (ESRD) secondary to IgA nephropathy is rarely reported in the literature. This case is particularly noteworthy as it demonstrates the evolution from kidney-limited disease (IgAN) to systemic vasculitis (IgAV) in the same patient. Adult IgA vasculitis, particularly in patients with ESRD, is relatively uncommon and may present significant diagnostic challenges due to overlapping clinical features with common dialysis-related complications.

## Case presentation

A 51-year-old woman with ESRD secondary to biopsy-proven IgA nephropathy on thrice-weekly hemodialysis presented to the emergency department with acute right shoulder and neck pain, progressive fatigue, and abdominal pain. Her medical history included hypertension, type 2 diabetes mellitus, hyperlipidemia, heart failure with preserved ejection fraction, sick sinus syndrome status post permanent pacemaker implantation, hypothyroidism, ischemic stroke, and prior upper gastrointestinal bleeding from a duodenal ulcer treated endoscopically.

On admission, vital signs were stable, and the patient was afebrile. Physical examination revealed conjunctival pallor, abdominal distension with mild epigastric tenderness, and bilateral lower-extremity edema. Notably, no rash was present on initial examination.

Laboratory studies demonstrated severe anemia with hemoglobin 6.6 g/dL (baseline: 9-10 g/dL), thrombocytopenia (95×10⁹/L), elevated blood urea nitrogen, and hyperkalemia consistent with missed dialysis sessions. The degree of thrombocytopenia was not sufficient to account for the observed purpura, supporting a non-thrombocytopenic mechanism consistent with small-vessel vasculitis. Stool was positive for occult blood, and melena was subsequently observed, confirming upper gastrointestinal bleeding. Upper endoscopy with gastric biopsies demonstrated reactive gastropathy without evidence of *Helicobacter pylori* infection or vasculitis. These findings are consistent with patterns of gastrointestinal involvement in IgA vasculitis, where mucosal inflammation may occur in the absence of histopathologic evidence of vasculitis. The patient received packed red blood cell transfusions and was started on high-dose proton pump inhibitor therapy.

Abdominal ultrasound and computed tomography revealed gallbladder wall thickening with pericholecystic edema and mild ascites, initially raising concern for acalculous cholecystitis. However, hepatobiliary scintigraphy demonstrated normal gallbladder function, and these findings were attributed to volume overload rather than acute cholecystitis.

**Figure 1:**
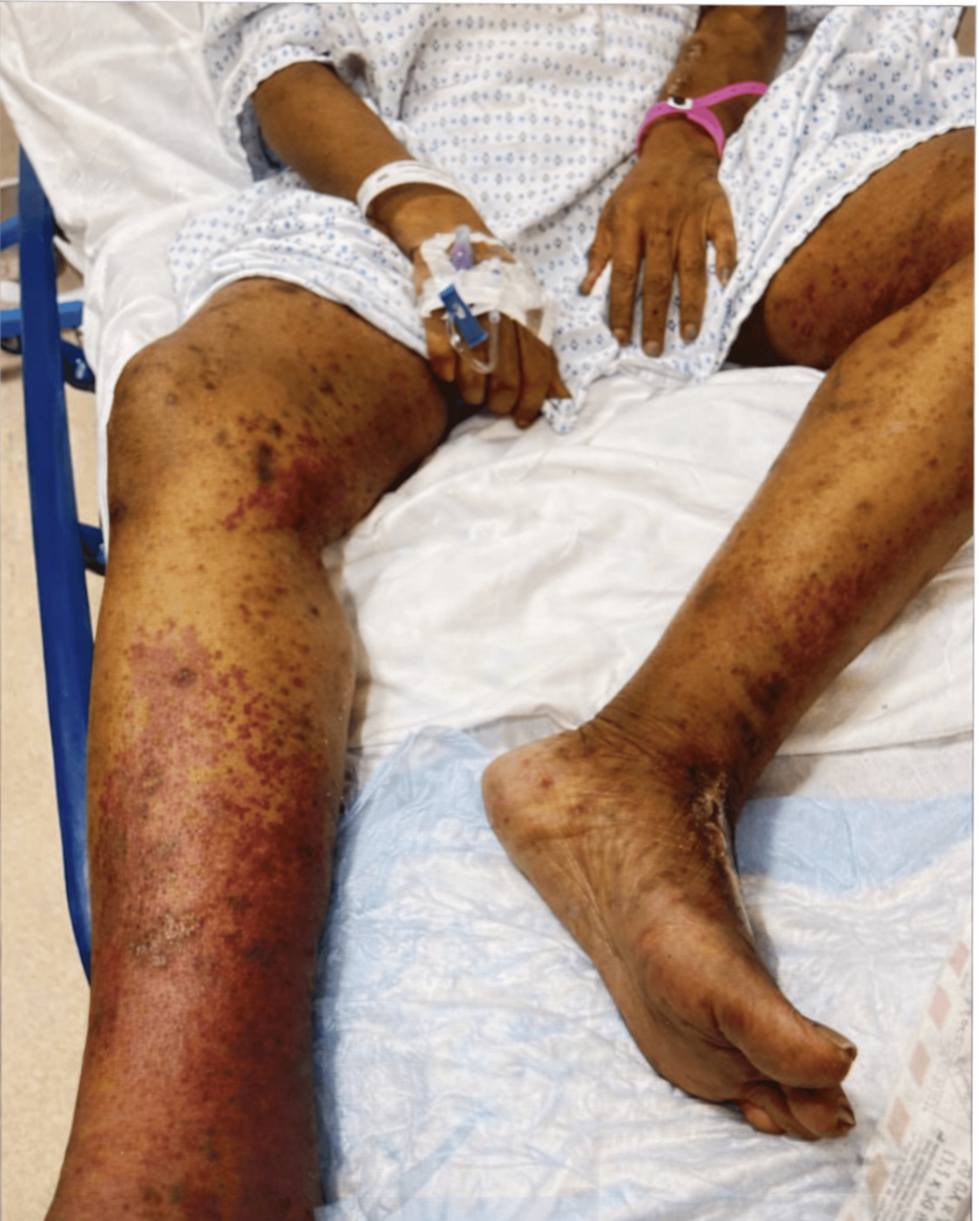
Palpable purpura involving bilateral lower extremities. Diffuse non-blanching violaceous macules and patches involving the bilateral lower extremities, with areas of confluence, consistent with small-vessel vasculitis.

On hospital day three, the patient developed a painful, palpable purpuric rash on both lower extremities with associated arthralgias involving the knees and ankles. The rash was characterized by non-blanching, violaceous macules and patches with areas of confluence, predominantly involving the bilateral lower extremities and extending proximally. Given the classic presentation of palpable purpura, abdominal pain, arthralgias, and her history of IgA nephropathy, there was high clinical suspicion for IgA vasculitis (Figure 1).

Dermatology and rheumatology consultations were obtained. Skin biopsy of the purpuric lesions demonstrated leukocytoclastic vasculitis on routine histology, characterized by a perivascular neutrophilic infiltrate with nuclear debris (leukocytoclasia), fibrin deposition, and extravasated erythrocytes. Direct immunofluorescence (DIF) revealed perivascular IgA, IgM, and C3 deposition. These findings, in conjunction with the patient's palpable purpura, abdominal pain, arthralgias, and history of IgA nephropathy, confirmed the diagnosis of IgA vasculitis.

An extensive autoimmune workup had been performed at an outside facility, revealing an antinuclear antibody (ANA) screen >1:2560 (homogeneous pattern), with negative myeloperoxidase (MPO) antibodies, proteinase 3 antibodies, histone antibodies, C-ANCA, P-ANCA, Sjögren syndrome antibodies, anti-Smith antibodies, anti-dsDNA antibodies, beta-2 microglobulin antibodies, and cardiolipin antibodies. Complement levels (C3/C4) were normal. ADAMTS13 activity was normal, and the disseminated intravascular coagulation (DIC) workup was negative. Notably, the patient had a documented history of vasculitis symptoms that improved with discontinuation of hydralazine and recurred upon re-exposure to the medication, suggesting possible drug-induced vasculitis.

The patient was treated with high-dose systemic corticosteroids (methylprednisolone 1 mg/kg daily), resulting in rapid improvement of the rash, arthralgias, and abdominal symptoms. A subsequent biopsy of a persistent lesion did not demonstrate features of active vasculitis, likely reflecting treatment response and temporal evolution of lesions following corticosteroid therapy. Hydralazine was permanently discontinued. Dialysis prescriptions were adjusted to address volume overload and electrolyte abnormalities. Her hemoglobin stabilized without further transfusion requirements, and gastrointestinal bleeding resolved with medical therapy. She was discharged on a corticosteroid taper with close outpatient follow-up with nephrology, rheumatology, and gastroenterology. At one-month follow-up, the rash had completely resolved, and no recurrent bleeding or systemic symptoms were reported.

## Discussion

This case illustrates several important clinical and pathophysiologic concepts regarding the relationship between IgA nephropathy and IgA vasculitis. IgA vasculitis and IgA nephropathy are now understood to represent different manifestations of the same disease spectrum rather than separate entities [4]. Both conditions share a common pathogenic mechanism involving galactose-deficient IgA1 (Gd-IgA1).

While IgAN and IgAV share pathogenic mechanisms, the development of systemic vasculitis in patients with established ESRD from IgA nephropathy is rarely reported. Most patients present with either kidney-limited disease (IgAN) or systemic disease with renal involvement (IgAV) at the time of initial diagnosis. The evolution from one form to the other in the same patient is uncommon and not well-characterized in the literature.

Our patient's presentation is particularly unique because she had documented IgA nephropathy that progressed to ESRD, and then years later developed systemic manifestations of IgA vasculitis. This suggests that the underlying IgA-mediated immune dysregulation persisted despite progression to ESRD and may have been triggered by external factors such as medication exposure.

The diagnosis of IgA vasculitis requires demonstration of leukocytoclastic vasculitis with IgA deposition on direct immunofluorescence [6,7]. In our patient, skin biopsy showed classic leukocytoclastic vasculitis on routine histology, and direct immunofluorescence (DIF) revealed perivascular IgA, IgM, and C3 deposition. The differential diagnosis in this case included uremia-related platelet dysfunction, drug-induced leukocytoclastic vasculitis, infection, and cholesterol embolization. However, the presence of palpable purpura, systemic symptoms, and confirmatory immunofluorescence findings supported the diagnosis of IgA vasculitis.

The classic tetrad of IgA vasculitis includes - (1) palpable purpura (100% of cases), typically on lower extremities and buttocks; (2) arthralgias or arthritis (61%), especially affecting knees and ankles; (3) gastrointestinal involvement (53%), manifesting as abdominal pain, nausea, and bleeding; and (4) glomerulonephritis (70%) [8,9]. Our patient demonstrated all four classic features. The palpable purpura appeared on hospital day three, which is typical, as skin manifestations may not be present at initial presentation. The purpuric rash is characteristically non-thrombocytopenic and results from leukocytoclastic vasculitis of dermal capillaries [2].

Gastrointestinal involvement in adult IgAV is common (53% of cases) and typically presents with abdominal pain (99%), intestinal bleeding (31%), and diarrhea (26%) [9]. Severe complications such as intestinal perforation and mesenteric ischemia can occur but are rare [9]. In our patient, the initial presentation with abdominal pain and gastrointestinal bleeding was concerning for IgAV, though the diagnosis was not confirmed until the characteristic rash appeared.

An important aspect of this case is the potential role of hydralazine as a trigger for vasculitis [10]. Our patient had documented improvement of vasculitis symptoms with hydralazine discontinuation and recurrence upon re-exposure, strongly suggesting drug-induced disease. While the temporal relationship suggests hydralazine may have acted as a potential trigger, this association remains interpretative, as ANCA-negative drug-induced IgA vasculitis is uncommon. It is possible that our patient had an underlying predisposition to IgA-mediated disease (evidenced by her IgA nephropathy), and hydralazine exposure triggered the systemic manifestations. Corticosteroids are the first-line therapy for adult IgA vasculitis with significant systemic involvement [11]. Our patient responded well to high-dose methylprednisolone with rapid resolution of cutaneous and systemic symptoms.

The management decision in this case was influenced by the suspected drug-induced component. Discontinuation of the offending drug is the mainstay of therapy for drug-induced vasculitis [12]. Given the clear temporal relationship with hydralazine exposure, permanent discontinuation of this medication was essential. The patient's prior improvement after hydralazine cessation suggested that a corticosteroid taper alone might be sufficient, without requiring long-term steroid-sparing immunosuppressive agents.

This case highlights the diagnostic complexity of vasculitis in ESRD patients. The initial presentation with anemia, abdominal pain, and gastrointestinal bleeding could easily be attributed to common dialysis-related complications such as uremic gastritis, arteriovenous malformations, or medication-related bleeding. Notably, gastrointestinal biopsy findings did not demonstrate vasculitis, which is consistent with prior studies indicating that histopathologic confirmation is not always present despite clinically significant gastrointestinal involvement in IgA vasculitis. The delayed appearance of palpable purpura on hospital day three underscores the importance of maintaining a high index of suspicion for systemic vasculitis even when classic cutaneous findings are not initially present.

Furthermore, the interpretation of serologic markers in ESRD patients can be challenging. Elevated ANA titers may be seen in up to 20-30% of dialysis patients without autoimmune disease. The absence of vasculitis on subsequent biopsy may reflect the dynamic nature of cutaneous lesions and the effects of corticosteroid therapy, emphasizing the importance of early biopsy in suspected cases. The absence of ANCA antibodies in our patient, despite the clinical suspicion for drug-induced vasculitis, required careful integration of clinical, histopathologic, and immunofluorescence findings to arrive at the correct diagnosis.

## Conclusions

This case demonstrates the evolution from kidney-limited IgA nephropathy to systemic IgA vasculitis in an adult patient with ESRD on maintenance hemodialysis. Adult IgA vasculitis should be considered in dialysis-dependent patients presenting with unexplained rash, arthralgias, and gastrointestinal bleeding. Recognition of IgAV in ESRD patients is challenging due to overlapping comorbidities, but is critical for timely immunosuppressive therapy. Multidisciplinary management can lead to favorable outcomes despite diagnostic complexity. While this case supports a possible link between IgA nephropathy and subsequent systemic vasculitis, broader generalizability remains limited, and further studies are needed to better characterize this relationship.
